# Comparative Evaluation of Temporomandibular Joint Parameters in Unilateral and Bilateral Cleft Lip and Palate Patients Using Cone-Beam CT: Focus on Growing vs. Non-Growing Subjects

**DOI:** 10.3390/healthcare12161563

**Published:** 2024-08-07

**Authors:** Ahmed Z. Abdelkarim, Ahmed A. Almeshari, Duygu Celik Ozen, Ayman R. Khalifa, Nader N. Rezallah, Suayip Burak Duman, Sonam Khurana

**Affiliations:** 1 Division of Oral and Maxillofacial Radiology, College of Dentistry, The Ohio State University, Columbus, OH 43210, USA; 2 Department of Oral and Maxillofacial Surgery and Diagnostic Sciences, Faculty of Dentistry, Najran University, Najran 66441, Saudi Arabia; aaalmeshari@nu.edu.sa; 3 Department of Oral and Maxillofacial Radiology, Faculty of Dentistry, Inonu University, 44210 Malatya, Turkey; duygu.celik@inonu.edu.tr (D.C.O.); burak.duman@inonu.edu.tr (S.B.D.); 4 Department of Community Dentistry, College of Dentistry, Gulf Medical University, Ajman 4181, United Arab Emirates; dr.aymankhalifa@gmu.ac.ae; 5 Division of Oral and Maxillofacial Radiology, College of Dentistry, City University Ajman, Ajman 18484, United Arab Emirates; n.nabil@cu.ac.ae; 6 Department of Oral and Maxillofacial Pathology, Radiology and Medicine, NYU College of Dentistry, New York, NY 10010, USA; sonam.khurana@nyu.edu

**Keywords:** cleft lip and palate, CBCT, temporomandibular joint

## Abstract

Background: Morphological differences in the temporomandibular joint (TMJ) are crucial for the treatment of patients with cleft lip and palate (CLP). This study aims to evaluate and compare the TMJ parameters in patients with unilateral and bilateral CLP across growing and non-growing age groups using cone-beam computed tomography (CBCT). Methods: CBCT records from 57 patients (23 males and 34 females) aged 6–50 years with a diagnosed unilateral or bilateral CLP were analyzed. Patients were categorized into four groups: growing unilateral (UGCLP), growing bilateral (BGCLP), non-growing unilateral (UNGCLP), and non-growing bilateral (BNGCLP). Measurements of TMJ parameters, including the mandibular fossa, articular eminence inclination, joint spaces, and roof thickness of the glenoid fossa, were conducted using CBCT images. Results: Significant differences were observed in the anterior joint space (AJS) and the roof of the glenoid fossa (RGF) between growing and non-growing unilateral cleft patients. Additionally, significant discrepancies were found in the articular eminence angle when comparing the cleft and non-cleft sides within the unilateral growing group. No significant differences were observed in TMJ parameters between the right and left sides among bilateral cleft patients. Conclusions: The study highlights distinct TMJ morphological differences between growing and non-growing patients with CLP, emphasizing the importance of age-specific considerations in the treatment planning and growth monitoring of these patients.

## 1. Introduction

Cleft lip and palate (CLP) are prevalent craniofacial anomalies marked by an underdeveloped maxilla, impaired growth, and morphological and functional alterations of the facial bones [1]. These anomalies arise from multifactorial etiologies, including genetic factors such as IRF6, ch8q24, VAX1, FGFR2, and BMP4, which affect the development of the maxilla and mandible. Environmental factors such as maternal smoking, alcohol consumption, and certain medications during pregnancy can also contribute to these anomalies [2]. Facial morphology is established between the fourth and tenth weeks of gestation through the fusion of five prominences. By the end of the seventh week, mesodermal migration facilitates the fusion of the maxillary process with the intermaxillary process, potentially resulting in cleft lip and palate. The cleft may affect only the lip or extend deeply into the alveolar process and secondary palate [3]. Embryologically, insufficient neural crest cell migration prevents the maxillary and medial nasal elevations from merging, leading to a cleft lip, while failure of the lateral palatine processes to connect and fuse results in a cleft palate [4]. The most common facial clefts can be unilateral, bilateral, complete, or incomplete [3].

Despite varying opinions across studies regarding the dimensions of the temporomandibular joint (TMJ), orthognathic surgeries have been prescribed for 25% of patients with cleft lip and palate [5]. Additionally, some researchers have reported a reduced maxillary sinus volume in these patients [6]. Craniofacial asymmetry between the affected and unaffected sides has been observed in unilateral clefts (ULCs) [7]. Tziavaras et al. (2010) identified morphological alterations in facial bones distant from the cleft region. Their study found significant differences in the mandibular body and gonial angle between cleft and non-cleft sides, indicating broader craniofacial impacts of cleft lip and palate beyond the immediate cleft area [8]. Similarly, Lin et al. (2015) found that the mandibular body on the cleft side was shorter and the gonial angle was larger compared to the non-cleft side, although no significant differences were noted in the superior anterior and superior posterior joint spaces between the cleft and non-cleft sides [9].

Similarly, the primary surgery of the lip and palate results in healed fibrous tissue that restricts maxillary growth, often leading to displacement, malocclusion, and facial asymmetry [10]. However, the differences in the TMJ between growing and non-growing individuals with clefts have not been adequately evaluated and remain unclear. The TMJ is crucial and encompasses several clinical issues. It is an important factor in treatment planning and growth monitoring, particularly in patients with unilateral or bilateral cleft lip and palate, as it plays a key role in achieving good occlusion and maintaining a stable stomatognathic system [11].

CBCT has proven valuable in analyzing TMJs due to its relatively low radiation dose, affordability, and excellent spatial resolution of multi-planar images compared to conventional CT. It effectively eliminates the disadvantages of superimposition and distortion seen in conventional radiology [12,13]. Despite its limitations in soft tissue imaging, CBCT is preferred for assessing TMJs in various cases of temporomandibular joint disorders (TMDs) because of its high dimensional accuracy for osseous structures [13,14]. Additionally, CBCT provides satisfactory volume measurements that are comparable to standard multi-detector row computed tomography (MDCT) [15].

To the best of our knowledge, no previous studies have specifically examined growing or non-growing individuals with clefts. Therefore, this study aims to analyze various TMJ measurements and parameters using CBCT and compare them between growing and non-growing patients with unilateral or bilateral clefts.

## 2. Materials and Methods

### 2.1. Method of Data Collection

CBCT images were acquired at the Department of Oral and Maxillofacial Radiology, Faculty of Dentistry, Malatya Inönü University, using the Newtom 5G XL system (Patterson Companies, Inc., Saint Paul, MN, USA). Informed consent was obtained from all patients or their caregivers, permitting the use of their data for scientific research. The study included 57 patients with cleft lip and palate (CLP), consisting of 23 males and 34 females, who were selected through convenience sampling; the patients were chosen based on the availability of their CBCT records and willingness to participate in the study.

Two board-certified radiologists evaluated the CBCT data of the CLP sample, which was divided into four groups based on the cleft type (unilateral or bilateral) and age (growing or non-growing). The classification into growing and non-growing was determined using specific age and sex-related growth cessation criteria, such as males typically ceasing growth by age 18 and females continuing to grow up to age 17. The growing cleft group consisted of 36 subjects aged 6–17 years, with 15 in the unilateral-growing cleft lip and palate (UGCLP) subgroup and 21 in the bilateral-growing cleft lip and palate (BGCLP) subgroup. The non-growing cleft group included 21 subjects aged 18–50 years, with 14 having unilateral clefts and 7 having bilateral clefts.

### 2.2. TMJ Analysis

CBCT images were acquired using the ILUMA Ultra CT Scanner system, KODAK 9000 3D System, and IMTEC (Imaging Corporation, Ardmore, OK, USA). Each scan was performed with a voxel size of 0.023 mm, a scan duration of 40 s, and a 16-bit grayscale resolution ranging from 31,744 to 45,811 shades of gray, producing a 1:1 scale image with a CT slice thickness of 0.28 mm. The images were analyzed on an HP 15-inch flat square LED monitor with a screen resolution of 1280 × 1080 and fixed contrast and brightness settings to ensure optimal visualization.

**Figure 1:**
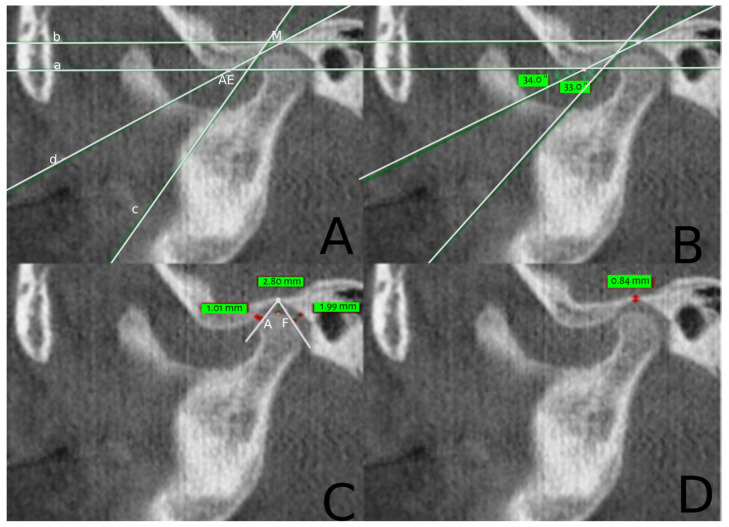
Images showing the various parameters of the temporomandibular joint. (**A**) Image showing mandibular fossa (MF), articular eminence (AE), and lines a, b, c, and d; (**B**) image showing articular eminence inclination AEI and X and Y angles; (**C**) image showing anterior joint space (AJS), posterior joint space (PJS), and superior joint space (SJS); (**D**) image showing thickness of the roof of glenoid fossa (RGF).

The primary aim of this research was to examine the central sections of the condyle in both the lateral and medial regions. To accomplish this, a pilot study was undertaken, making use of two reference studies for measuring the mandibular fossa [16,17]. The parameters assessed included the mandibular fossa, articular eminence inclination, anterior, superior, and posterior joint spaces, as well as the thickness of the glenoid fossa’s roof. Prior to taking measurements, specific points and lines were identified to ensure consistency. The measurements were conducted using NewTom’s NNT software v 3.10 and its measurement tool, and the data were recorded in an Excel spreadsheet for further statistical analysis (Table 1 and Figure 1).

### 2.3. Statistical Analysis

A sample size calculation was executed based on data from a pilot study comprising five patients in each of the control growing and non- growing unilateral groups. To ensure a power of 80%, a minimum sample size of seven patients was deemed necessary. Ten de-identified cases were randomly sampled, and two observers (A.Z.A. and A.A.A.) were tasked with analyzing the intra- and inter-rater reliability of the measurements. This process was iterated five times, with a washout period of five weeks between repetitions. The intraclass correlation coefficient (ICC) derived from the measurements was utilized to evaluate observation reliability, with the goal of achieving an ICC of at least 0.80 through repeated trials [18].

Statistical analyses were conducted employing appropriate methods for the gathered data. The appropriate methods for the gathered data included both descriptive and inferential statistics. Descriptive statistics summarized the central tendency and variability, while inferential statistics evaluated the significance of group differences using the Mann–Whitney U test for independent samples and the Wilcoxon rank test for within-patient comparisons. This ensured a robust analysis suitable for the study’s sample size and data type. Statistical significance was set at *p* < 0.05. All analyses were conducted using R Studio version 4.2.2.

## 3. Results

A total of 57 study subjects were included, comprising 34 females (59.6%) and 23 males (40.4%). The median age of the subjects was 14.00 years (interquartile range: 9.00, 19.00 years). The subjects were categorized into four groups: growing bilateral [21 subjects (36.8%)], growing unilateral [15 subjects (26.3%)], non-growing bilateral [7 subjects (12.3%)], and non-growing unilateral [14 subjects (24.6%)]. Given the small and non-homogeneous study groups, detailed characteristics including the age and standard deviation (SD) for each group are provided below:

- Growing bilateral cleft group: mean age = 12.3 years, SD = 3.4;
- Growing unilateral cleft group: mean age = 10.8 years, SD = 4.1;
- Non-growing bilateral cleft group: mean age = 28.7 years, SD = 6.2;
- Non-growing unilateral cleft group: mean age = 32.5 years, SD = 5.7.

### 3.1. Source of Data

The CBCT scans of patients with unilateral and bilateral cleft lip and palate, obtained from the Department of Oral and Maxillofacial Radiology, Faculty of Dentistry, Malatya Inonu University, between 2010 and 2022, were evaluated based on predetermined criteria and included in the study. The necessary ethical committee approval was obtained for the de-identified CBCT scans of patients with and without cleft lip and palate. The study protocol was approved by the Inonu University Non-interventional Clinical Research Ethics Committee on 19 July 2023 with the decision number 2023/4815. The study was conducted in accordance with the principles of the Declaration of Helsinki, revised in 2003.

Patients aged between 6 and 50 years, who had undergone preoperative cone-beam computed tomography (CBCT) imaging at the oral and maxillofacial radiology department and were pre-diagnosed with cleft lip and palate, were included in the study. CBCT images acquired using standard parameters and the complete Digital Imaging and Communications in Medicine (DICOM) files were used. The study excluded individuals with a history of trauma, orthognathic surgical repair, airway abnormalities, or pathologies such as laryngomalacia, severe asthma, chronic obstructive pulmonary disease (COPD), or known or suspected craniofacial syndromes associated with airway abnormalities (e.g., Pierre–Robin sequence) that could confound the assessment of cleft lip and palate. No individuals with cleft lip and palate underwent preoperative orthodontic intervention.

### 3.2. Unilateral Cleft Patient

#### 3.2.1. Comparison of Cleft-Side Parameters between Growing and Non-Growing Patients

**Figure 2:**
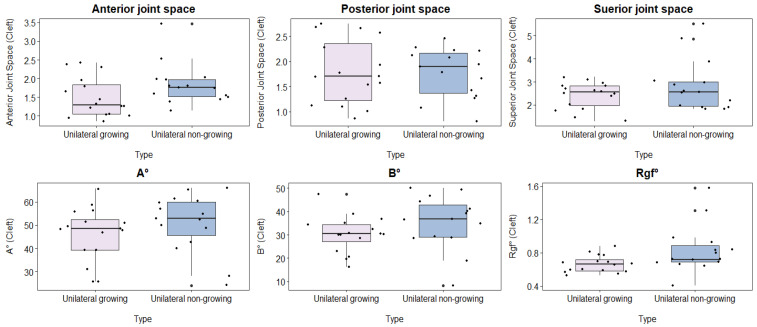
Comparison of the cleft-side parameters among the unilateral cleft cases between growing and non-growing patients (Articular eminence inclination X angle (A°), Articular eminence inclination Y angle (B°), and Roof of glenoid fossa (Rgf°)).

Significant differences were observed in the anterior joint space (AJS) (*p* = 0.046) and roof of the glenoid fossa (RGF) (*p* = 0.019). However, no significant differences were found in the posterior joint space (PJS), superior joint space (SJS), articular eminence inclination (AEI) X-angle, or AEI Y-angle measurements (Table 2; Figure 2).

#### 3.2.2. Comparison of Non-Cleft-Side Parameters between Growing and Non-Growing Patients

**Figure 3:**
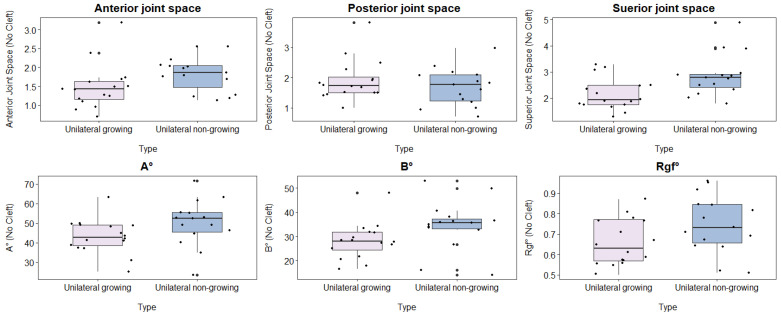
Comparison of the non-cleft-side parameters among the unilateral cleft cases between growing and non-growing patients (Articular eminence inclination X angle (A°), Articular eminence inclination Y angle (B°), and Roof of glenoid fossa (Rgf°)).

Significant differences were observed in the following parameters: AJS (*p* = 0.031), SJS (*p* = 0.011), AEI X angle (*p* = 0.04), and AEI Y angle (*p* = 0.011). However, no statistically significant differences were found in the PJS and RGF measurements (Table 3; Figure 3).

### 3.3. Bilateral Cleft Patients

#### 3.3.1. Comparison of the Left-Side Parameters among Bilateral Cleft Cases between Growing and Non-Growing Patients with a Bilateral Cleft

**Figure 4:**
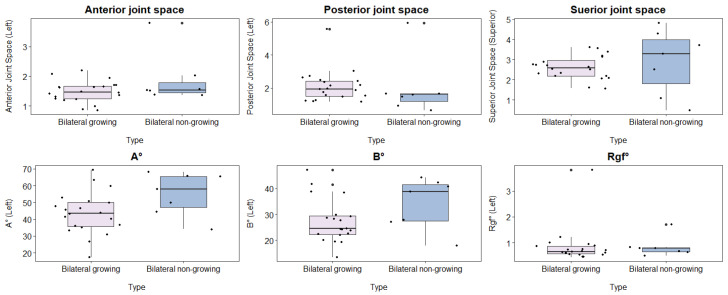
Comparison of the left-side parameters among the bilateral cleft cases between growing and non-growing patients with a bilateral cleft (Articular eminence inclination X angle (A°), Articular eminence inclination Y angle (B°), and Roof of glenoid fossa (Rgf°)).

A comparison between bilateral clefts that were growing and non-growing on the left side showed no discrepancies in any of the parameters (as presented in Table 4 and Figure 4).

#### 3.3.2. Comparison of the Right-Side Parameters among Bilateral Cleft Cases between Growing and Non-Growing Patients with a Bilateral Cleft

**Figure 5:**
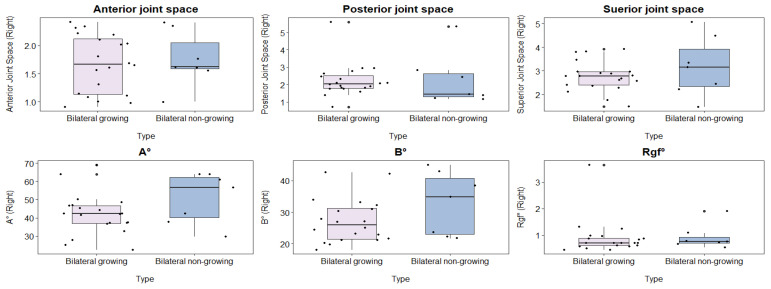
Comparison of the right-side parameters among the bilateral cleft cases between growing and non-growing patients with a bilateral cleft (Articular eminence inclination X angle (A°), Articular eminence inclination Y angle (B°), and Roof of glenoid fossa (Rgf°)).

A comparison of bilateral clefts on the right side, both growing and non-growing, showed no significant variations in any of the factors listed in Table 5 and depicted in Figure 5.

### 3.4. Comparison of Parameters between Growing and Non-Growing Unilateral Cleft Patients

#### 3.4.1. Growing Patients

When comparing the parameters of the cleft and non-cleft sides within growing patients, most parameters did not show significant differences (Table 6) except for the following:

- SJS: significant difference (*p* = 0.027);
- AEI X (A°) angle: significant difference (*p* = 0.039);
- AEI Y angle (B°): significant difference (*p* = 0.004).

#### 3.4.2. Non-Growing Patients

The comparison between the cleft and non-cleft sides within non-growing patients showed no significant differences (Table 7). In summary, while most parameters did not show significant differences between the cleft and non-cleft sides within both growing and non-growing groups, notable differences were observed in specific parameters among the growing patients.

### 3.5. Comparison of Right and Left Sides in Bilateral Cleft Patients

#### 3.5.1. Comparison between the Parameters of the Right and Left Sides within Bilateral Growing Patients

There were no significant differences found in the parameters between both groups (Table 8).

#### 3.5.2. Comparison between the Parameters of the Right and Left Sides within Bilateral Non-Growing Patients

No significant differences in any of the parameters could be appreciated (Table 9).

## 4. Discussion

Clefts in the facial area are common congenital abnormalities affecting the lips, palate, and alveolar bone. Patients with cleft lip and palate face challenges like facial deformities, malocclusions, and difficulties with nutrition, respiration, hearing, and speech. While dental and craniofacial research is extensive [6,19] the temporomandibular joint (TMJ) has received little attention. This study aimed to compare the TMJ measurements in individuals with unilateral and bilateral clefts.

This research assessed the joint spaces in individuals with UCLP, comparing those who were growing to those who were not. The findings demonstrated that there was a substantial difference between the two groups, particularly in the anterior joint space, which was larger in the non-cleft patients. Moreover, among the UCLP patients with non-cleft sides, a substantial difference was noted between the growing and non-growing subjects, specifically in the anterior and superior joint spaces. However, for patients with bilateral clefts, no significant variations were observed in any of the parameters on either side.

In the group with a unilateral cleft, considerable variations were noticed in the superior joint space (SJS) and the inclination of the articular eminence (X and Y angles) among growing subjects. Previous research by Patil et al. [20], Rodrigues et al. [21], and Fraga et al. [22] discovered significant distinctions in the anterior joint space (AJS) and posterior joint space (PJS) in UCLP and unilateral non-cleft patients, which aligns with our study’s outcomes. These studies suggested that the anterior joint spaces were smaller than the posterior joint spaces. Similarly, Lin et al. [9] utilized the Pullinger formula to evaluate condylar concentricity and assessed the sizes of the AJS and PJS. Their findings indicated that joint concentricity is influenced by the joint size, and the disparities between the AJS and PJS are due to varying joint sizes [23]. In their study, the AJS was smaller than the PJS, with differences observed when comparing cleft and non-cleft sides, which is consistent with our study results. Patil et al. [20] also scrutinized anterior, posterior, and superior joint spaces, concluding that these spaces were largest in non-cleft cases and smallest in UCLP patients.

In patients with UCLP, we discovered considerable disparities in the anterior joint space (AJS) and superior joint space (SJS) on the non-cleft side between individuals who were growing and those who were not. In contrast to the results of Lin et al. [9], our study found that the AJS was smaller than the posterior joint space (PJS). Furthermore, the sizes of the AJS, PJS, and SJS were greatest in non-cleft cases and smallest in UCLP cases. However, there are few studies that have examined these parameters in patients with bilateral clefts, and our study revealed no substantial differences between the growing and non-growing sides in these patients.

Facial asymmetry in patients with unilateral cleft lip and palate (UCLP) has been linked to a distorted nasomaxillary complex [8]. According to Laspos et al. [24], no significant mandibular asymmetry is observed, and the possibility is more so due to cranial base anomalies. Various studies have offered inconsistent opinions, as the mandible is not directly affected by the cleft, indicating a possible multifactorial etiology. Veli et al. [25] found no asymmetry in unilateral clefts, except for elongation of the coronoid process on the cleft side. However, our study revealed asymmetry in patients with UCLP and symmetrical growth patterns in those with bilateral clefts. Celikogu et al. [26] also reported shorter ramal and condylar heights on the cleft side. Our findings of asymmetry in UCLP are supported by Kurt et al. [27], who noted higher condylar height in cleft patients compared to non-cleft patients, suggesting asymmetry. Paknahad et al. [28] found that condylar ramus mandibular asymmetry was more common in patients with unilateral cleft lip and palate (UCLP) than in those with bilateral cleft lip and palate (CLP). Similarly, Neiswanger et al. [7] observed fluctuating asymmetry in ear length measurements in individuals with UCLP compared to their unaffected relatives.

We subsequently assessed the thickness of the glenoid fossa’s roof. No substantial contrast was identified between the cleft and non-cleft cohorts, whether unilateral or bilateral. These results align with Celikoglu et al.’s [26] findings, who also reported no significant discrepancies in this parameter. In a similar vein, Ejima et al. [29] concluded that the thickness of the glenoid fossa is unaffected by condylar morphology in both cleft and non-cleft groups. We also evaluated the articular eminence inclination (X and Y) on the non-cleft side of unilateral cleft patients and non-cleft individuals in both growing and non-growing subjects. A more significant inclination was noticed on the non-cleft side in non-growing patients, but no variation was observed on the cleft side. Additionally, no significant contrast in the X and Y angles was discovered in bilateral cleft patients between the growing and non-growing groups.

In working with subjects who have unilateral clefts, a noticeable discrepancy was observed between the cleft and non-cleft sides, while no such difference was present in those with bilateral clefts. Particularly, a heightened inclination of the articular eminence was observed on the non-cleft side in unilateral subjects. These findings align with those of Talaeipour et al. [30], who reported a significantly lower articular eminence angle in subjects with cleft lip and palate (CLP) compared to the controls. To our knowledge, the specific parameters we examined have not been extensively studied in the literature, as most research on articular eminence angulation has focused on temporomandibular disorder (TMD) cases rather than cleft cases. A related study was conducted by Ucar et al. [31], who investigated the position of the mandibular condyle and temporomandibular fossa in adolescent patients with bilateral cleft lip and palate (BCLP) compared to well-matched controls without any cleft using cone-beam computed tomography (CBCT). Their study found similar values for all parameters except for the condylar angulation on the right side, with the BCLP group showing less angulation than the controls. Additionally, the condylar volume was slightly lower in the BCLP group on both sides compared to the control group. Overall, the positions of the mandibular condyle and temporomandibular fossa were similar in patients with BCLP and the non-cleft control group [31].

Clinicians need to evaluate the size disparities between the dental arches before commencing treatment to guarantee proper alignment and ideal occlusion. Orthodontists in particular must be aware of tooth size differences in patients with CLP to choose the optimal treatment and accurately predict outcomes. For instance, larger molars on the cleft side demand more attention and a tailored approach compared to smaller incisors. Understanding these variations enables clinicians to devise treatment plans that cater to these specific characteristics, thereby achieving optimal results. In our recent study, we found no correlation between asymmetries in growing and non-growing bilateral clefts and non-clefts with tooth size and malocclusion. A meta-analysis conducted by Antonarakis et al. [32] concluded that non-syndromic unilateral CLP patients tend to have smaller maxillary central incisors, lateral incisors, and first molars on the cleft side, while their mandibular teeth are generally larger.

Previous research has investigated the growth patterns of various structures in the heads and necks of CLP subjects, yielding mixed results. For instance, a study by Abdelkarim et al. [33] found that growing CLP patients displayed more variances in the shape of the hyoid bone, suggesting that both growth and the cleft palate condition affect its morphology. However, another study by Abdelkarim et al. [34] on the pharyngeal airway revealed no significant differences between growing cleft patients and control groups. Nevertheless, significant linear and angular differences were observed in the nasal cavity and maxillary sinus on the cleft side of the non-growing cleft group [34]. Unilateral CLP is often associated with mandibular deformities that are typically overlooked in traditional studies, which primarily focus on deformities of the lip, nose, and maxilla. These mandibular deformities are characterized by a shortened mandibular body, posterior rotation, and retrusion. Studies suggest that patients with unilateral CLP may exhibit mandibular asymmetry and deformity, as indicated by differences in the hemi-mandible volume and linear/angular measurements between the cleft and non-cleft sides. This implies that mandibular deformities can worsen with growth and time, mirroring the affected maxilla. A study by Lo et al. [35] confirmed that mandibular asymmetry and deformity are indeed measurable in patients with unilateral CLP.

Monitoring changes in mandibular dysmorphology over time, especially after surgery, is essential for evaluating treatment outcomes and planning future interventions. According to Celikoglu et al. [26], understanding these deformities can improve cleft palate treatment protocols, even though the conditions in the UCLP and BCLP groups differ. Their study found comparable craniofacial measurements and mandibular volumes between the two groups [26].

The discrepancies in our results might be attributed to the types of radiographs and evaluation methods used, as well as the various treatment modalities recommended for cleft cases. Skeletal and bony changes in the temporomandibular joint (TMJ) following splint therapy in cleft patients may help reduce temporomandibular disorders (TMDs) and mandibular deviations [36,37]. Furthermore, the absence of early treatment approaches may lead to an altered TMJ anatomy and increased TMD incidence in cleft patients [38].

Our research provides valuable insights that can help clinicians refine treatment strategies for individuals with clefts. However, our study has limitations. We were unable to evaluate subjects with TMDs, which could have enriched our findings and comparisons with other studies. Additionally, our sample was limited to a single tertiary care center, and a more diverse population could have been studied in a national survey.

## 5. Limitations

Despite its contributions, this study has several limitations. The sample size and diversity were restricted to a single tertiary care center, which may not represent the broader population of CLP patients. Future research should aim to include a larger and more diverse cohort to enhance the generalizability of the findings. Additionally, the study provides a snapshot of TMJ parameters at specific time points, lacking longitudinal data. Studies tracking changes over time, especially pre- and post-surgery, would offer deeper insights into the progression and management of TMJ issues in CLP patients. Addressing these limitations will help refine treatment protocols and improve care for individuals with cleft lip and palate.

## 6. Conclusions

This study evaluates temporomandibular joint (TMJ) parameters in patients with unilateral and bilateral cleft lip and palate (CLP) using cone-beam computed tomography (CBCT). By comparing growing and non-growing groups, we identified significant differences in TMJ morphology that are crucial for clinical management.

Key findings include significant variations in the anterior joint space (AJS) and the roof of the glenoid fossa (RGF) in growing unilateral cleft patients compared to non-growing ones. In the non-growing unilateral cleft group, no significant asymmetry was found. Among the growing patients, three out of six parameters did not reach statistical significance, indicating that the asymmetry detected in growing patients diminishes after growth ceases. Bilateral cleft patients showed no significant differences in TMJ parameters between sides. These results highlight the need for age-specific treatment strategies for CLP patients, emphasizing early intervention for growing individuals. The use of CBCT has been instrumental in accurately assessing TMJ structures, aiding clinical decisions.

In summary, understanding the growth-related changes in TMJ morphology is essential for optimizing treatment and outcomes for CLP patients. Future research should focus on longitudinal studies to further explore TMJ development and its clinical implications. Notably, the observed reduction in asymmetry post-growth cessation warrants further investigation to tailor age-appropriate interventions effectively.

## Figures and Tables

**Table 1:** Parameters determined on CBCT images of TMJ before measurement.

Parameters	Description
Mandibular fossa (MF)	The uppermost point in the mandibular fossa pattern was taken as the reference point (Figure 1A).
Articular eminence (AE)	The lowest point on the articular eminence pattern was drawn into four lines (Figure 1A): Line parallel to the Frankfurt plane passing through the AE point Line parallel to the Frankfurt plane passing through the MF point Line tangent to the largest possible surface of the posterior slope of the articular eminence Line through MF and AE points
Articular eminence inclination (AEI)	5. The inclination in the anteroposterior direction was obtained using 2 angles (Figure 1B): 6. X angle: the angle formed by the intersection of line a and line c 7. Y angle: the angle formed by the intersection of line a and line b
Temporomandibular joint space	8. For the temporomandibular joint space measurement, the MF point was connected with a tangent to the most prominent points on the anterior (A) and posterior (P) faces of the condyle (Figure 1C).
Anterior joint space (AJS)	9. The perpendicular distance measured from tangent point A to the glenoid fossa (Figure 1C).
Posterior joint space (PJS)	10. The perpendicular distance measured from the tangent point P to the glenoid fossa (Figure 1C).
Superior joint space (SJS)	11. The perpendicular distance measured from the superior prominent point of the condyle head to the MF point (Figure 1C).
Thickness of the roof of glenoid fossa (RGF)	12. It was measured as the vertical distance between the MF point and the intracranial fossa (Figure 1D).

**Table 2:** Comparison of the cleft-side parameters among the unilateral cleft cases between growing and non-growing patients.

Variable	Growing *n* = 16	Non-Growing *n* = 15	*p*-Value *
Anterior joint space (mm)	1.29 [1.05, 1.83]	1.76 [1.52, 1.98]	0.046
Posterior joint space (mm)	1.70 [1.23, 2.35]	1.89 [1.36, 2.16]	0.937
Superior joint space (mm)	2.55 [1.97, 2.83]	2.56 [1.94, 3.01]	0.45
Articular eminence inclination X angle (A°)	48.50 [39.30, 52.47]	52.90 [45.70, 60.00]	0.14
Articular eminence inclination Y angle (B°)	30.40 [27.18, 34.47]	36.80 [29.00, 42.70]	0.1
Roof of glenoid fossa (Rgf°)	0.66 [0.58, 0.72]	0.72 [0.69, 0.88]	0.019

* Mann–Whitney U test, mm = millimeter.

**Table 3:** Comparison of the non-cleft-side parameters among the unilateral cleft cases between growing and non-growing patients.

Variable	Growing *n* = 16	Non-Growing *n* = 15	*p*-Value *
Anterior joint space (mm)	1.42 [1.15, 1.64]	1.87 [1.48, 2.05]	0.031
Posterior joint space (mm)	1.73 [1.50, 2.02]	1.77 [1.23, 2.09]	0.5
Superior joint space (mm)	1.93 [1.76, 2.50]	2.78 [2.41, 2.92]	0.011
Articular eminence inclination X angle (A°)	42.90 [38.57, 49.05]	52.60 [45.50, 55.45]	0.04
Articular eminence inclination Y angle (B°)	28.10 [24.32, 31.75]	35.60 [33.20, 37.35]	0.011
Roof of glenoid fossa (Rgf°)	0.63 [0.57, 0.77]	0.73 [0.66, 0.84]	0.072

* Mann–Whitney U test, mm = millimeter.

**Table 4:** Comparison of the left-side parameters among the bilateral cleft cases between growing and non-growing patients with a bilateral cleft.

Variable	Growing *n* = 20	Non-Growing *n* = 7	*p*-Value *
Anterior joint space (mm)	1.46 [1.25, 1.66]	1.53 [1.45, 1.79]	0.361
Posterior joint space (mm)	1.94 [1.52, 2.44]	1.62 [1.21, 1.66]	0.184
Superior joint space (mm)	2.58 [2.19, 2.95]	3.28 [1.79, 4.00]	0.376
Articular eminence inclination X angle (A°)	43.40 [35.67, 50.10]	57.90 [47.00, 65.55]	0.068
Articular eminence inclination Y angle (B°)	24.50 [22.27, 29.32]	38.70 [27.40, 41.50]	0.135
Roof of glenoid fossa (Rgf°)	0.65 [0.58, 0.87]	0.78 [0.66, 0.80]	0.455

* Mann–Whitney U test, mm = millimeter.

**Table 5:** Comparison of the right-side parameters among the bilateral cleft cases between growing and non-growing patients with a bilateral cleft.

Variable	Growing *n* = 20	Non-Growing *n* = 7	*p*-Value *
Anterior joint space (mm)	1.67 [1.13, 2.12]	1.62 [1.58, 2.06]	0.698
Posterior joint space (mm)	2.04 [1.79, 2.50]	1.44 [1.30, 2.62]	0.406
Superior joint space (mm)	2.78 [2.39, 2.96]	3.15 [2.33, 3.91]	0.543
Articular eminence inclination X angle (A°)	42.20 [36.98, 46.70]	56.60 [40.10, 62.30]	0.175
Articular eminence inclination Y angle (B°)	26.00 [21.50, 31.27]	34.80 [23.00, 40.70]	0.15
Roof of glenoid fossa (Rgf°)	0.70 [0.60, 0.89]	0.75 [0.70, 0.93]	0.506

* Mann–Whitney U test, mm = millimeter.

**Table 6:** Comparison between the parameters of the cleft and non-cleft sides within the unilateral growing patients.

Variable	Cleft Side *n* = 16	Non-Cleft Side *n* = 16	*p*-Value *
Anterior joint space (mm)	1.29 [1.05, 1.83]	1.42 [1.15, 1.64]	0.83
Posterior joint space (mm)	1.70 [1.23, 2.35]	1.73 [1.50, 2.02]	0.19
Superior joint space(mm)	2.55 [1.97, 2.83]	1.93 [1.76, 2.50]	0.027
Articular eminence inclination X angle (A°)	48.50 [39.30, 52.47]	42.90 [38.57, 49.05]	0.039
Articular eminence inclination Y angle (B°)	30.40 [27.18, 34.47]	28.10 [24.32, 31.75]	0.004
Roof of glenoid fossa (Rgf°)	0.66 [0.58, 0.72]	0.63 [0.57, 0.77]	0.88

* Mann–Whitney U test, mm = millimeter.

**Table 7:** Comparison between the parameters of the cleft and non-cleft sides within the unilateral non-growing patients.

Variable	Cleft Side *n* = 15	Non-Cleft Side *n* = 15	*p*-Value *
Anterior joint space (mm)	1.76 [1.52, 1.98]	1.87 [1.48, 2.05]	0.86
Posterior joint space (mm)	1.89 [1.36, 2.16]	1.77 [1.23, 2.09]	0.29
Superior joint space (mm)	2.56 [1.94, 3.01]	2.78 [2.41, 2.92]	0.82
Articular eminence inclination X angle (A°)	52.90 [45.70, 60.00]	52.60 [45.50, 55.45]	0.43
Articular eminence inclination Y angle (B°)	36.80 [29.00, 42.70]	35.60 [33.20, 37.35]	0.37
Roof of glenoid fossa (Rgf°)	0.72 [0.69, 0.88]	0.73 [0.66, 0.84]	0.42

* Mann–Whitney U test, mm = millimeter.

**Table 8:** Comparison between the parameters of the right and left sides within bilateral growing patients.

Variable	Right Side *n* = 20	Left Side *n* = 20	*p*-Value *
Anterior joint space (mm)	1.67 [1.13, 2.12]	1.46 [1.25, 1.66]	0.1
Posterior joint space (mm)	2.04 [1.79, 2.50]	1.94 [1.52, 2.44]	0.34
Superior joint space (mm)	2.78 [2.39, 2.96]	2.58 [2.19, 2.95]	0.19
Articular eminence inclination X angle (A°)	42.20 [36.98, 46.70]	43.40 [35.67, 50.10]	0.41
Articular eminence inclination Y angle (B°)	26.00 [21.50, 31.27]	24.50 [22.27, 29.32]	0.96
Roof of glenoid fossa (Rgf°)	0.70 [0.60, 0.89]	0.65 [0.58, 0.87]	0.2

* Mann–Whitney U test, mm = millimeter

**Table 9:** Comparison between the parameters of the right and left sides within bilateral non-growing patients.

Variable	Right Side *n* = 7	Left Side *n* = 7	*p*-Value *
Anterior joint space (mm)	1.62 [1.58, 2.06]	1.53 [1.45, 1.79]	0.92
Posterior joint space (mm)	1.44 [1.30, 2.62]	1.62 [1.21, 1.66]	0.5
Superior joint space (mm)	3.15 [2.33, 3.91]	3.28 [1.79, 4.00]	0.4
Articular eminence inclination X angle (A°)	56.60 [40.10, 62.30]	57.90 [47.00, 65.55]	0.13
Articular eminence inclination Y angle (B°)	34.80 [23.00, 40.70]	38.70 [27.40, 41.50]	0.237
Roof of glenoid fossa (Rgf°)	0.75 [0.70, 0.93]	0.78 [0.66, 0.80]	0.08

* Mann–Whitney U test, mm = millimeter.

## Data Availability

The data presented in this study are available on request from the corresponding author. The data are not publicly available due to privacy.
